# Proteomics Indicators of the Rapidly Shifting Physiology from Whole Mountain Pine Beetle, *Dendroctonus ponderosae* (Coleoptera: Curculionidae), Adults during Early Host Colonization

**DOI:** 10.1371/journal.pone.0110673

**Published:** 2014-10-31

**Authors:** Caitlin Pitt, Jeanne A. Robert, Tiffany R. Bonnett, Christopher I. Keeling, Jörg Bohlmann, Dezene P. W. Huber

**Affiliations:** 1 Ecosystem Science and Management Program, University of Northern British Columbia, Prince George, BC, Canada; 2 Michael Smith Laboratories, University of British Columbia, Vancouver, BC, Canada; Natural Resources Canada, Canada

## Abstract

We developed proteome profiles for host colonizing mountain pine beetle adults, *Dendroctonus ponderosae* Hopkins (Coleoptera: Curculionidae). Adult insects were fed in pairs on fresh host lodgepole pine, *Pinus contorta* Dougl. ex Loud, phloem tissue. The proteomes of fed individuals were monitored using iTRAQ and compared to those of starved beetles, revealing 757 and 739 expressed proteins in females and males, respectively, for which quantitative information was obtained. Overall functional category distributions were similar for males and females, with the majority of proteins falling under carbohydrate metabolism (glycolysis, gluconeogenesis, citric acid cycle), structure (cuticle, muscle, cytoskeleton), and protein and amino acid metabolism. Females had 23 proteins with levels that changed significantly with feeding (*p<0.05*, FDR<0.20), including chaperones and enzymes required for vitellogenesis. In males, levels of 29 proteins changed significantly with feeding (*p<0.05*, FDR<0.20), including chaperones as well as motor proteins. Only two proteins, both chaperones, exhibited a significant change in both females and males with feeding. Proteins with differential accumulation patterns in females exhibited higher fold changes with feeding than did those in males. This difference may be due to major and rapid physiological changes occurring in females upon finding a host tree during the physiological shift from dispersal to reproduction. The significant accumulation of chaperone proteins, a cytochrome P450, and a glutathione S-transferase, indicate secondary metabolite-induced stress physiology related to chemical detoxification during early host colonization. The females' activation of vitellogenin only after encountering a host indicates deliberate partitioning of resources and a balancing of the needs of dispersal and reproduction.

## Introduction

The mountain pine beetle (MPB), *Dendroctonus ponderosae* Hopkins (Coleoptera: Curculionidae), is a member of the large subfamily Scolytinae (∼6,000 species) contained within the extremely large (∼60,000 species) and diverse weevil family [1]–[2]. Like all other weevils, MPB is an herbivore and it spends most of its life cycle closely associated with its host plant [3]. In the case of MPB, the host plant can be any of the pine species native to western North America [4] as well as novel pine hosts in an expanding range [5]–[6]. This intimate association with its host has resulted in a large number of traits that allow MPB to survive, and even to thrive, in the often-hostile environment of its host pine's tissues [7]–[8].

While MPB is an important ecological factor in western North American pine forests [9] – ranging from Mexico to central British Columbia, and from the west coast to the Black Hills of South Dakota – it has, in recent years, moved into an outbreak state of unprecedented dimensions. In British Columbia alone, this insect impacted up to 53% of merchantable pine by 2012 [10] causing tens of billions of dollars in economic damage. This outbreak is predicted to have long-term extreme effects on forest ecology and carbon cycling [11] and has devastated local silvicultural economies.

Recently, MPB has breached the historical barrier of the Canadian Rocky Mountains and has begun to spread in the lodgepole pine (*Pinus contorta* Douglas ex Louden) and hybrid lodgepole pine x jack pine (*Pinus banksiana* Lamb.) forests of northwestern Alberta. Since the MPB is known to be able to attack and kill almost every pine species [4] – and even occasionally spruces [12] – in its native range, it is not surprising that it has recently been found to be spreading and successfully reproducing in wild stands of pure jack pines [5]. The MPB is now considered an invasive pest that threatens boreal jack pine forests, and other pine species as well [6], ranging from central Alberta through the Great Lakes region and to the eastern seaboard of Canada and the United States of America.

We are interested in the critical host colonization phase of the beetle's life cycle when it must cooperatively invade and kill its host tree so that it can later successfully reproduce [3]–[4]. While most of the insect's life cycle is cryptic – spent under the bark of its brood tree – the adults can spend several days in a foraging dispersal flight [13] in the summer during which they find and colonize new host pines for reproduction. During the dispersal phase the insects – especially the females, which are the pioneering sex – search for new susceptible host trees in a mixed landscape that includes a variety of nonhosts [14]–[15] and non-susceptible individuals of the host species. Dispersing beetles do not feed, and so this phase is fraught with hazards including reduction in energy available for reproductive provisioning [16], encounters with predators or other antagonists, and potentially inclement weather. When insects find a suitable host, colonization is coordinated by aggregation and antiaggregation pheromones [17] that allow the insects and their symbiotic fungi [18]–[19] to overcome the complex and copious host defenses [20]–[22] and complete their reproductive cycle.

Host colonization begins immediately following the MPB's dispersal flight. We postulate that insect physiology must rapidly shift from supporting flight and host search towards survival when exposed to host defenses and commencing with reproductive activities. The host-colonizing beetles and their associated fungi must tolerate, detoxify or in other ways overcome host defenses and eventually kill the host prior to reproducing. Using expressed sequence tag (EST) data for the MPB [23], combined with a proteomics approach, we have observed a number of proteins that indicate a shift towards reproduction and may be involved in the stress physiology of insect survival during the initial period of host colonization. This investigation of the total and differentially expressed proteomes of adult male and female MPB during the host colonization phase of their life cycle will provide candidates for further study to better understand, and to perhaps better manage, this ecologically and economically important forest insect pest [5]–[6].

## Materials and Methods

### Beetles

Lodgepole pine bolts infested with MPB were field collected from an outbreak area near Penticton, British Columbia, in May 2010 (49°25′60″N; 119°27′30″W). Doug Bateman Logging collected the bolts from a company harvest; no further permission was required and no endangered or protected species were involved. Immediately after collection the ends of the bolts were sealed with molten paraffin wax to prevent drying during experiments. Bolts were placed in vented plastic containers and stored at ambient outdoor temperatures. In early July, young MPB adults began to emerge and were collected daily. Sex was determined by examining the 7^th^ abdominal tergite [24]. Emerged adults were stored in Petri dishes with a lightly moistened Kimwipe at 4°C. Beetles were stored for a maximum of ten days prior to use.

### Feeding treatments

On the 26^th^ and 27^th^ of July 2010, control beetles were placed individually in 1.5 mL tubes with perforated lids and kept in the dark for 24 hours at room temperature prior to flash-freezing in liquid nitrogen and storage at −80°C. Simultaneously, randomly paired female and male adults were placed into holes drilled into the phloem of freshly collected and waxed bolts of a healthy lodgepole pine tree collected near Prince George, BC (53°58′12″N; 122°52′18″W). Holes were spaced roughly 10 cm apart. The female was inserted first, followed by a male; a metal screen was stapled over the hole to prevent escape. Logs were set upright and beetles were left to feed at room temperature for 24 hours. Logs were then dissected to remove live individuals that had produced visible frass (boring dust and feces, indicating active phloem tissue colonization), and the sex of each recovered beetle was confirmed. Recovered beetles were immediately flash-frozen in liquid nitrogen and stored at −80°C.

### Protein extraction

Eight frozen adult MPB were used per extraction and treatment (control females, fed females, control males, fed males) and each extraction and treatment was replicated four times with different beetles. Samples were thawed on ice for 5 minutes then homogenized with 500 µl TCA buffer (trichloroacetic acid, Sigma) containing 15% TCA and 1% DTT (dithiothreitol, Fisher Scientific) by weight [25]. The samples were homogenized six times for 1 min each time at 1,500 cycles per min (2000 Geno/Grinder, Spex SamplePrep) interspersed with 3 min incubation on ice. The samples were transferred to new tubes and incubated on ice for 30 min. Samples were centrifuged at 18,000xg for 10 min at 4°C and the supernatant was removed. The pellets were resuspended in 1 mL ice-cold acetone and incubated on ice for 5 min. The acetone wash was repeated four times. The washed pellets were air dried for 10 min to allow the acetone to evaporate completely, then suspended in 1 mL urea:thiourea buffer containing 6 M Urea (Fisher Scientific) and 1 M thiourea (Fisher Scientific) in 100 mM Tris-Cl pH 8.0 (Ultrapure, Invitrogen). A final centrifugation at 18,000xg for 10 min at room temperature removed any remaining insoluble debris. The supernatant containing total insect protein was removed and stored at −80°C prior to subsequent iTRAQ proteome analysis. Protein concentration and quality was confirmed by a Coomassie (Bradford) Protein Assay Kit (Fisher Scientific) and Experion Pro260 Chip analysis (Biorad).

### iTRAQ analysis

Samples were processed at the University of Victoria Genome British Columbia Proteomics Centre (Victoria, British Columbia, Canada) for proteome quantification and analysis. Analysis used eight-plex isobaric tags for relative and absolute quantification (iTRAQ) [26]–[27]. Samples were analyzed as sets of two eight-plexes. The first multiplex included four biological replicates of control female-derived proteins and four biological replicates of fed female-derived proteins. The eight-plex of male protein samples followed in an identical manner. Experimental conditions were reported as follows: Protein concentrations were determined using a Bradford protein assay (Sigma). Samples (100 µg of each) were precipitated overnight in acetone at 4°C followed by resolubilzation in 0.5 M TEAB, 0.2% SDS. Proteins were reduced with TCEP and alkylated with MMTS. Proteins were then in solution digested with trypsin (Promega) and labeled with the appropriate iTRAQ label. Labeled peptides were then combined and separated by strong cation exchange HPLC. SCX fractions containing peptides were then reduced in volume by speed-vac and analyzed by LC-MS/MS. The length of the reverse gradient used was 2 hours per HPLC strong cation exchange fraction. Samples were analyzed by reversed phase nanoflow (300 nL/min) HPLC with nano-electrospray ionization using a quadrupole time-of-flight mass spectrometer (QStar pulsar i, Applied Biosystems) operated in positive ion mode.

### Data analysis

Data were analyzed using ProteinPilot Software v3.0 (Applied Biosystems) with the Paragon algorithm [28]. Analytical parameters were as follows: Cys alkylation: MMTS; digestion: trypsin; instrument: QSTAR ESI; and an Unused ProtScore threshold of >1.3 (95% protein confidence). Proteins were identified against the translated sequences from a MPB EST-derived transcriptome database [23].

Data were presented as ratios, normalized to one of the four biological control replicates for each sex, which was subsequently assigned a quantity of 1. Protein abundances were represented by average fold-change for each protein relative to control samples from untransformed iTRAQ ratios. Ratios were log_10_ transformed to obtain a normal distribution and a two-tailed independent samples *t*-test was conducted on the differences between starved control and fed treatment groups for each identified protein within each eight-plex. A Benjamini and Hochberg Correction (BH) was applied to the *p*-values to estimate the false discovery rate (FDR) [29]–[30]. Proteins were considered differentially expressed at *p<0.05* with an FDR<0.20 [31].

Proteins were manually annotated by their best BLASTx match to other insect proteins using their representative assembled EST sequences. Each quantitatively detected protein was investigated for its general functional role and subcellular location using UniProtKB [32] and the Panther Database [33]. The information was used to assign each protein to a general functional category (Tables S1 and S2 in File S1).

## Results

### Overall male and female proteome composition

Female and male samples yielded 757 and 739 proteins, respectively, for which quantitative information was obtained (Tables S1 and S2 in File S1). Proteins detected from females and males were designated by their EST library matches. 463 expressed proteins were shared between the sexes, leaving 294 and 276 proteins unique to the female and male proteomes, respectively.

**Figure 1:**
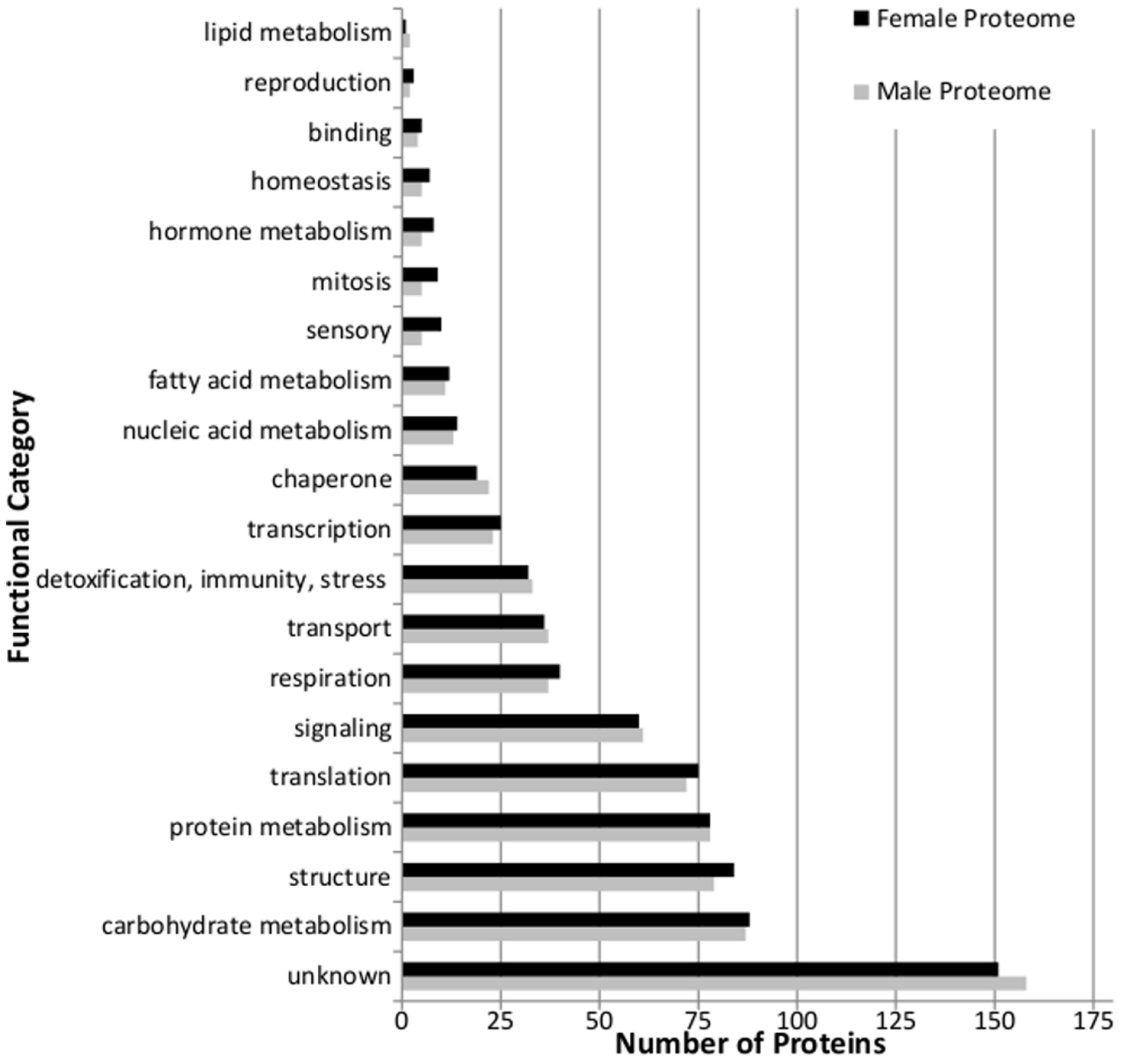
General functional proportions of all proteins. Detected proteins were divided into generalized functional groups based on gene ontology information from UNIprot.

All proteins were assigned to general functional categories using annotations provided by NCBI BLASTx and UNIprotKB (Figure 1). Besides those with unknown designations, the functional categories containing the most proteins were carbohydrate metabolism (11.6% in females, 11.8% in males; including enzymes involved in glycolysis, gluconeogenesis, and the citric acid cycle), structure (11.1% in females, 10.7% in males; proteins related to the cuticle, muscle, and cytoskeleton), protein and amino acid metabolism (10.3% in females, 9.7% in males), translation (9.9% in females and 10.6% in males; including ribosomal components and elongation factors), and signaling (7.9% in females, 8.3% in males; including enzymes affiliated with mediating cellular differentiation and development). In spite of the fact that only 61–63% of detected proteins were shared between the sexes, general proportions of proteins present in the functional groups differed little between females and males, perhaps suggesting common sources of precursors are being used for differing purposes.

**Figure 2:**
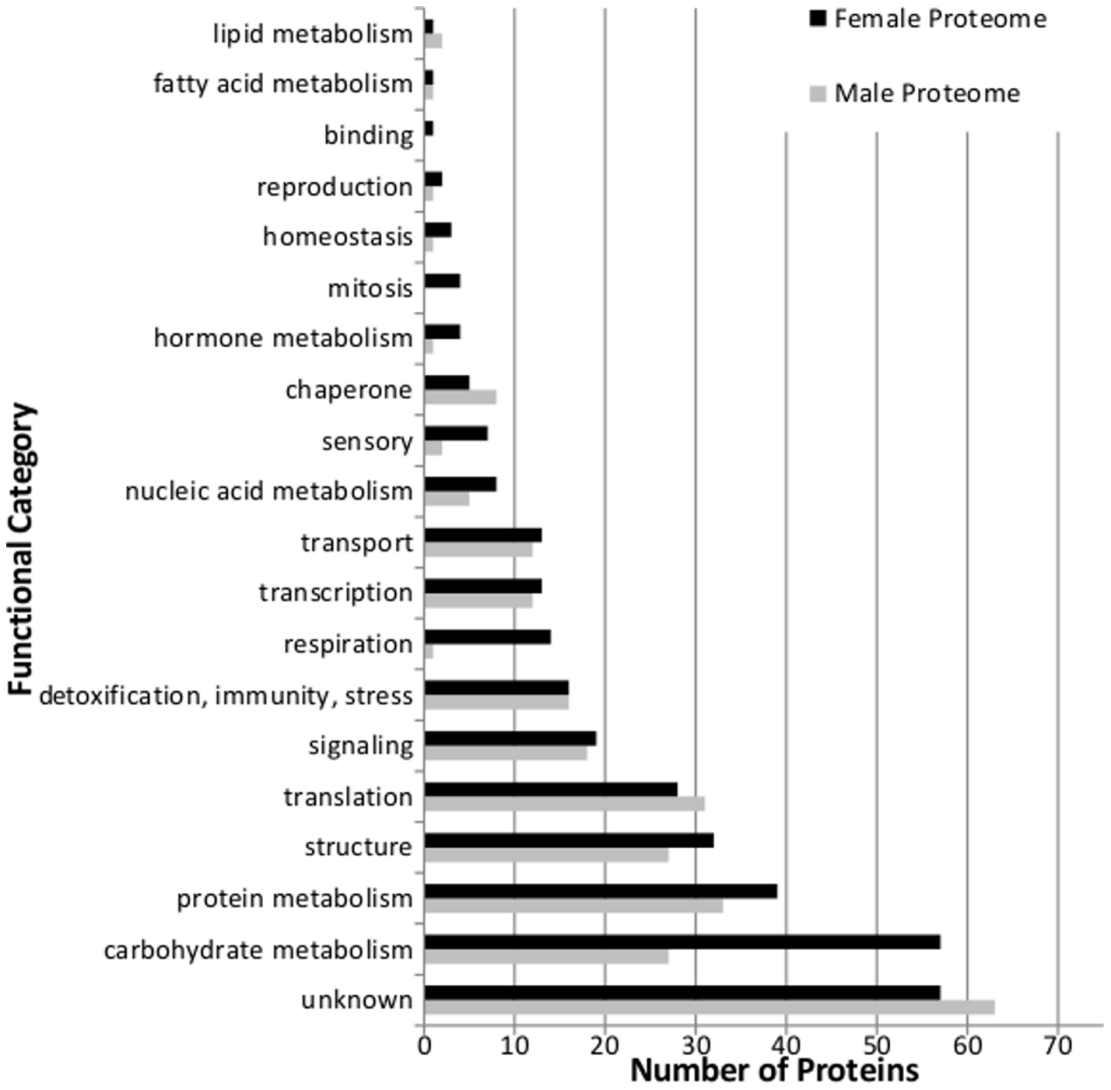
Functional distribution of detected proteins that were unique to each sex.

The general functional distributions of the detected proteins unique to each sex were similarly compared (Figure 2). Females had more proteins involved in protein metabolism, respiration, and mitosis than did males. Males expressed isoprenoid biosynthesis-related enzymes not found in females, namely HMG-CoA synthase (HMG-S, h_cluster_01168-1+2) and a protein similar to geranylgeranyl pyrophosphate synthase (GGPPS, h_cluster_11996-1)(Table S2 in File S1). The most obvious difference between males and females in this experimental context was in carbohydrate metabolism, where females expressed more than double the complement of different enzymes involved in this process than did males.

### Proteomic shifts following feeding

**Figure 3:**
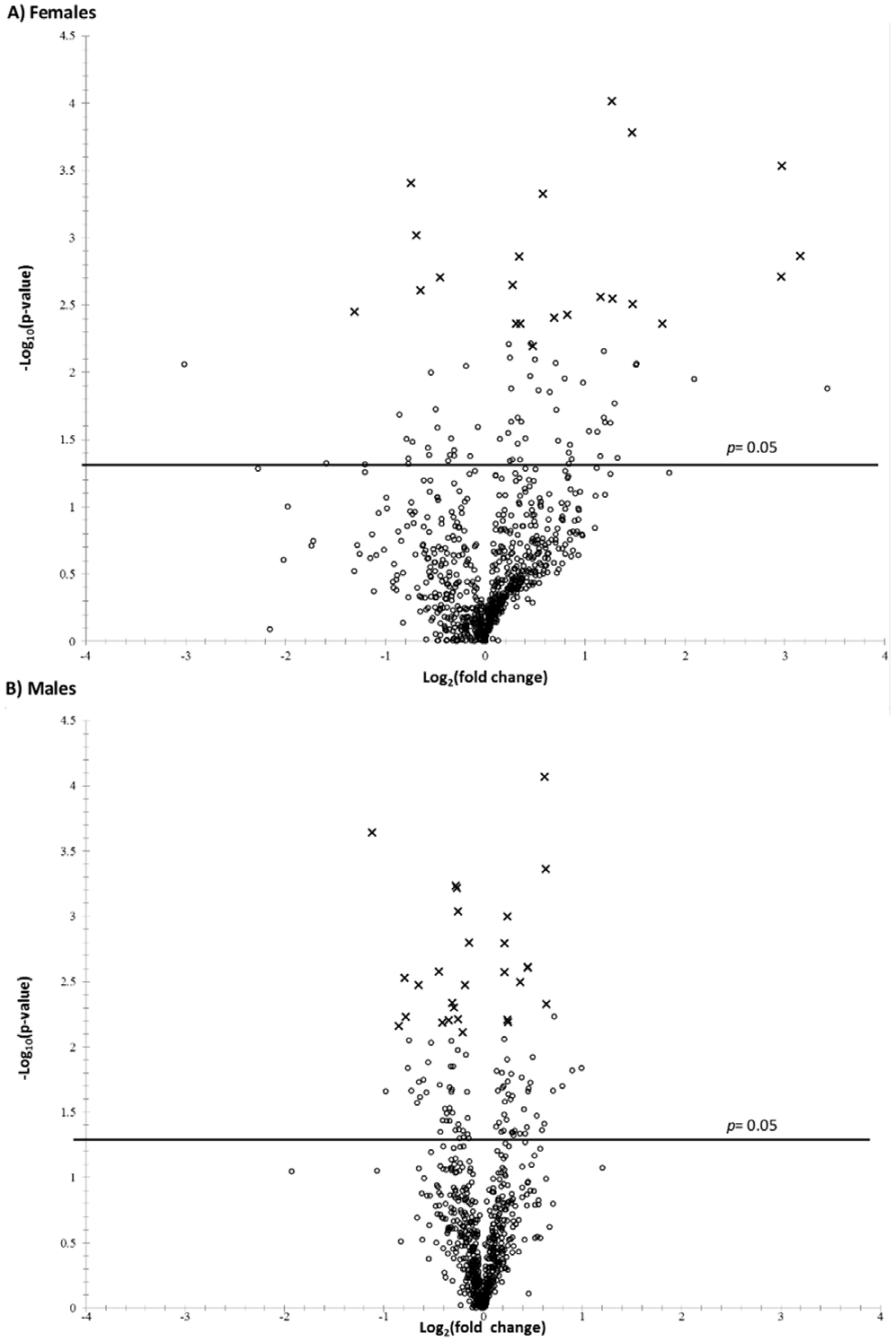
Volcano plots of iTRAQ protein quantification ratios in female (a) and male (b) iTRAQ runs. Points above the horizontal line represent proteins with significant (*p<0.05*) differences between starved and fed treatments, with points with FDR<0.2 marked as “x”.

The proportion of proteins that showed differential shifts in accumulation between treatments and controls, and the significance levels of those changes are depicted in volcano plots (Figure 3). Most detected proteins fell below the level of significance (*p<0.05*) when comparing treatments for either males or females against the male or female controls. The fold-changes of proteins showing significant differences in fed males versus control males are of lower magnitude (with one exception, all are under +/−2-fold) than those of female treatments (Tables 1 and 2).

**Table 1:** Proteins that showed significant changes in accumulation levels in females following feeding (*p<0.05*, FDR<0.2).

Translated EST contig ID^a^	EST/Genome Accession No.	BLASTx annotation	Subject	E-Value	Functional category	Fold change^b^	p-value
**s_cluster_03564+2**	**GT386951/YQE05171**	**TSA: Dendroctonus ponderosae Contig451, mRNA sequence**	**n/a**	**n/a**	**?**	**7.8**	**0.0003**
**h_cluster_16459-2+2**	**GT400641/YQE10688**	**TSA: Dendroctonus ponderosae Contig584, mRNA sequence**	**n/a**	**n/a**	**?**	2.4^*^	**0.0001**
**h_cluster_01569+1**	**GT469028/YQE04735**	**No Hits**	**n/a**	**n/a**	**?**	**−1.4**	**0.0020**
**h_cluster_12831+2**	**GT371138/YQE11261**	**similar to CG15105-PA, isoform A**	**tca:656122**	**3E-83**	**?**	**−1.6**	**0.0025**
**h_cluster_00498-1+3**	**GT323752/YQE11922**	**similar to CG8036-PB, isoform B; K00615 transketolase**	**tca:660692**	**0**	**carbohydrate metabolism**	**2.4**	**0.0028**
**h_cluster_02588-1+3**	**GT346060/YQE03281**	**G6pd; glucose-6-phosphate dehydrogenase; K00036 glucose-6-phosphate 1-dehydrogenase**	**tca:662934**	**0**	**carbohydrate metabolism**	**1.3**	**0.0043**
**h_cluster_00930-1+1**	**GT352910/YQE07729**	**similar to heat shock 70 kD protein cognate; K09490 heat shock 70 kDa protein 5**	**tca:659147**	**4E-141**	**chaperone**	3.4^*^	**0.0043**
**h_cluster_00722-1+1**	**GT484212/YQE07729**	**similar to ENSANGP00000012893; K09490 heat shock 70 kDa protein 5**	**nvi:100118379**	**0**	**chaperone**	2.8^*^	**0.0002**
h_cluster_00993-1+3	GT356404/YQE02814	similar to Probable cytochrome P450 6a14 (CYPVIA14), MPB CYP6DE1	tca:664473	9E-116	detoxification, immunity, stress response	1.8	0.0037
**h_cluster_00871-1+1**	**GT375000/YQE10157**	**similar to CG14661-PA**	**tca:663762**	**1E-33**	**hormone metabolism**	−2.5^*^	**0.0035**
h_cluster_03047+1	GT472768/YQE7451	similar to CG5840-PA, isoform A; K00286 pyrroline-5-carboxylate reductase	tca:664343	8E-99	protein metabolism	−1.6	0.0010
h_cluster_19511-3	GO487182/YQE09294	hypothetical protein (vitellogenin 6 precursor)	aag: AaeL_AAEL006138	2E-15	reproduction	8.9	0.0014
**h_cluster_01826-1+1**	**GT391859/YQE09293**	**similar to vitellogenin**	**tca:660042**	**2E-99**	**reproduction**	7.8^*^	**0.0019**
**h_cluster_00532-1+3**	**GT449473/YQE03475**	**similar to AGAP008724-PA; K02265 cytochrome c oxidase subunit Vb**	**tca:656612**	**5E-50**	**respiration**	**−1.7**	**0.0004**
**h_cluster_00098-1+1**	**GT337131/YQE05782**	**similar to 40S ribosomal protein SA (p40) (34/67 kDa laminin receptor); K02998 small subunit ribosomal protein SAe**	**tca:658600**	**1E-99**	**translation**	**2.8**	**0.0031**
**h_cluster_00022+2**	**GT333266/YQE11710**	**similar to S3Ae ribosomal protein; K02984 small subunit ribosomal protein S3Ae**	**tca:656438**	**9E-121**	**translation**	**1.8**	**0.0062**
**h_cluster_07603+3**	**GT338829/YQE07229**	**similar to ribosomal protein S8; K02995 small subunit ribosomal protein S8e**	**tca:655685**	**2E-99**	**translation**	**1.6**	**0.0039**
**h_cluster_14581+1**	**GT489617/YQE02752**	**similar to ribosomal protein S6; K02991 small subunit ribosomal protein S6e**	**tca:656796**	**2E-101**	**translation**	**1.5**	**0.0005**
**h_cluster_09822-1**	**GT339811/n/a**	**similar to Ribosomal protein L18A CG6510-PA; K02882 large subunit ribosomal protein L18Ae**	**ame:409832**	**2E-73**	**translation**	**1.4**	**0.0064**
h_cluster_00223+3	GT402293/YQE04450	similar to CG9282-PA; K02896 large subunit ribosomal protein L24e	tca:654912	5E-36	translation	1.3	0.0014
h_cluster_18699+2	GT490858/YQE07111	similar to ribosomal protein L31; K02910 large subunit ribosomal protein L31e	tca:663175	1E-50	translation	1.2	0.0043
**h_cluster_02284-1+3**	**GT403942/YQE01291**	**similar to Vacuolar ATP synthase catalytic subunit A, osteoclast isoform (V-ATPase A subunit 2) (Vacuolar proton pump alpha subunit 2) (V-ATPase 69 kDa subunit 2) (Isoform HO68); K02145 V-type H+-transporting ATPase subunit A**	**tca:663322**	**0**	**transport**	**2.2**	**0.0028**
**h_cluster_17723-2-2**	**GO487586/YQE00120**	**similar to Vacuolar ATP synthase catalytic subunit A, osteoclast isoform (V-ATPase A subunit 2) (Vacuolar proton pump alpha subunit 2) (V-ATPase 69 kDa subunit 2) (Isoform HO68); K02145 V-type H+-transporting ATPase subunit A**	**tca:663322**	**7E-105**	**transport**	**1.2**	**0.0022**

Plain text represents proteins whose levels changed following feeding that were unique to the female iTRAQ runs. Those in bold were also present in males. Bold, underlined contig Ids signify proteins whose levels also changed significantly in males. Fold change values marked with an asterisk also changed significantly in the RNA-seq transcriptome.

a Suffix (+1, +2, +3, −1, −2, −3) indicates translation frame. Contig sequences available in File S1.

b Fold change is relative to control (starved) females. Values marked with an asterisk also changed significantly in the transcriptome (51).

**Table 2:** Proteins that showed significant changes in accumulation levels in males following feeding (*p<0.05*, FDR<0.2).

Translated EST contig ID^a^	EST/Genome Accession No.	BLASTx annotation	Subject	E-Value	Functional category	Fold change^b^	p-value
**h_cluster_00011-1+2**	**GT413372/YQE02827**	**similar to CG6084-PA, isoform A**	**tca:658019**	**8E-144**	**?**	1.6^*^	**0.0058**
f_cluster_c4596+1	n/a/YQE09455	No Hits	n/a	n/a	?	1.5	0.0004
**h_cluster_06285-1+1**	**GT360056/YQE02828**	**similar to CG6084-PA, isoform A**	**tca:657943**	**9E-124**	**?**	**1.2**	**0.0062**
**h_cluster_00437-1+1**	**GT424689/YQE03298**	**similar to cxpwmw03; K03522 electron transfer flavoprotein alpha subunit**	**tca:657777**	**1E-141**	**?**	**−1.1**	**0.0016**
**h_cluster_08736-1+1**	**GT327980/YQE04947**	**similar to alanine aminotransferase; K00814 alanine transaminase [EC:2.6.1.2]**	**api:100164899**	**4E-120**	**?**	**−1.2**	**0.0061**
h_cluster_09712-2	GT428841/YQE06399	No Hits	n/a	n/a	?	−1.2	0.0006
h_cluster_01584-2	GT321155/n/a	No Hits	n/a	n/a	?	−1.8	0.0069
s_cluster_03357+1	GT380648/YQE03583	No Hits	n/a	n/a	?	−2.2	0.0002
**f_cluster_c11478+2**	**n/a/YQE07184**	**similar to CG11661-PF, isoform F; K00164 2-oxoglutarate dehydrogenase E1 component [EC:1.2.4.2]**	**tca:662219**	**7E-163**	**carbohydrate metabolism**	**−1.1**	**0.0033**
**h_cluster_10171-3-3**	**GT377795/YQE07184**	**similar to CG4001-PB, isoform B; K00850 6-phosphofructokinase [EC:2.7.1.11]**	**tca:658327**	**2E-148**	**carbohydrate metabolism**	**−1.2**	**0.0046**
**f_cluster_c577+3**	**GT478810/YQE02618**	**similar to CG1516-PE, isoform E; K01959 pyruvate carboxylase subunit A [EC:6.4.1.1]; K01960 pyruvate carboxylase subunit B [EC:6.4.1.1]**	**tca:662701**	**0**	**carbohydrate metabolism**	**−1.7**	**0.0059**
**h_cluster_00722-1+1**	**GT484212/YQE07729**	**similar to ENSANGP00000012893; K09490 heat shock 70 kDa protein 5**	**nvi:100118379**	**0**	**chaperone**	1.6^*^	**0.0047**
**h_cluster_00159-1+2**	**GT369839/YQE10054**	**similar to CG9429-PA: calreticulin**	**tca:660439**	**9E-163**	**chaperone**	1.5^*^	**0.0001**
**h_cluster_00930-1+1**	**GT352910/YQE07729**	**similar to heat shock 70 kD protein cognate; K09490 heat shock 70 kDa protein 5**	**tca:659147**	**4E-141**	**chaperone**	1.4^*^	**0.0025**
**h_cluster_00442-1+3**	**GT437135/YQE06755**	**similar to CG8938-PA, isoform A, DpGSTs4**	**tca:662754**	**6E-62**	**detoxification, immunity, stress response**	**1.3**	**0.0032**
**h_cluster_01581-1+3**	**GT330637/YQE07531**	**similar to AMP dependent coa ligase**	**tca:655052**	**0**	**fatty acid metabolism**	−1.2^*^	**0.0009**
**h_cluster_00955-1+1**	**GT463172/YQE11663**	**similar to CG4703-PA; K00248 butyryl-CoA dehydrogenase [EC:1.3.99.2] (short-chain specific acyl-CoA dehydrogenase, mitochondrial)**	**tca:661684**	**0**	**fatty acid metabolism**	**−1.2**	**0.0050**
**h_cluster_00668-1+1**	**GT452594/YQE03432**	**similar to CG9360-PA (3-oxoacyl-[acyl-carrier-protein] reductase)**	**tca:658671**	**1E-63**	**fatty acid metabolism**	**−1.7**	**0.0030**
h_cluster_08348+3	GT323407/YQE08406	similar to Low molecular weight phosphotyrosine protein phosphatase 2 (Low molecular weight cytosolic acid phosphatase 2) (PTPase 2)	tca:657373	1E-63	protein metabolism	1.2	0.0027
h_cluster_02035+3	GT326460/YQE10953	similar to CG13340-PA (cytosol and leucine aminopeptidase)	tca:663523	0	protein metabolism	−1.3	0.0065
**h_cluster_01760+1**	**GT378898/YQE02609**	**similar to AGAP007123-PA (sarcosine dehydrogenase - mitochondrial)**	**tca:656849**	**0**	**protein metabolism**	**−1.6**	**0.0033**
h_cluster_01629-1+3	GT473612/YQE07881	similar to proteasome (prosome, macropain) subunit, alpha type 1; K02725 20S proteasome subunit alpha 6 [EC:3.4.25.1]	tca:655339	3E-121	protein metabolsim	1.2	0.0064
h_cluster_00049-2+1	GT317557/YQE01516	similar to ATP synthase delta chain, mitochondrial; K02134 F-type H+-transporting ATPase subunit delta [EC:3.6.3.14]	tca:656683	6E-51	respiration	−1.4	0.0026
**h_cluster_02330-1+3**	**GT376610/YQE08847**	**similar to CG2331-PA, isoform A**	**tca:661187**	**0**	**signaling**	**1.2**	**0.0010**
h_cluster_03460+2	GT328587/YQE07802	similar to CG14996-PB; calponin-like protein Chd64	tca:663982	3E-86	structure	1.4	0.0024
**f_cluster_c3717+1**	**GT360252/YQE04803**	**similar to CG4376-PB, isoform B; K05699 actinin alpha**	**tca:661042**	**0**	**structure**	**−1.2**	**0.0077**
f_cluster_c3347-3	n/a/n/a	similar to Kinesin light chain (KLC); K10407 kinesin light chain	tca:655195	2E-98	structure	−1.3	0.0062
**h_cluster_13890-3**	**GT322908/YQE05135**	**similar to ribosomal protein S24e; K02974 small subunit ribosomal protein S24e**	**tca:655928**	**2E-49**	**translation**	**1.2**	**0.0016**
**h_cluster_06800+1**	**GT334993/YQE03706**	**GL10774 gene product from transcript GL10774-RA**	**dpe: Dper_GL10774**	**3E-117**	**translation**	**−1.2**	**0.0006**

Plain text represents proteins whose levels changed following feeding that were unique to the male iTRAQ runs. Those in bold were also present in females. Bold, underlined contig Ids signify proteins whose levels also changed significantly in females. Fold change values marked with an asterisk also changed significantly in the RNA-seq transcriptome.

a Suffix (+1, +2, +3, −1, −2, −3) indicates translation frame. Contig sequences available in File S1.

b Fold change is relative to control (starved) males. Values marked with an asterisk also changed significantly in the transcriptome (51).

A Benjamini and Hochberg correction was applied to the *p*-values to control for false discovery at the 0.20 level [31]. In females, 23 proteins showed expression differences in accumulation between starved and fed treatments (Table 1). Of these, 18 increased with feeding (from 1.2-fold to a maximum of 8.9-fold change) and five decreased (from -1.4-fold to -2.5 -fold change). Proteins changing in quantity with treatment included the following categories: carbohydrate metabolism, chaperone, detoxification/stress response, hormone metabolism, nucleic acid metabolism, protein metabolism, reproduction, respiration, sensory, signaling, structure, transcription, translation, and transport. Notably, vitellogenin proteins – glycolipoproteins that usually function as yolk precursors during oogenesis – exhibited some of the highest fold-change increases (7.8 and 8.9). Also increasing were two heat shock proteins and a cytochrome P450, identified as CYP6DE1. Those that showed significant decreases with feeding in females included a possible juvenile hormone binding protein, and proteins involved in proline biosynthesis and possibly odorant binding.

Of the proteins detected in males, 29 exhibited significant differences between fed and control treatments (Table 2). Twelve increased (ranging from 1.2-fold to 1.6-fold change) and 17 decreased (-1.1 to -2.2-fold change) with feeding. Proteins that changed with feeding treatment were classified in the following categories: carbohydrate metabolism, chaperone, detoxification/stress, fatty acid metabolism, homeostasis, protein metabolism, respiration, signaling, structure, translation, and transport. The heat shock proteins increasing in females also did so in males, as well as a glutathione S-transferase (GST, DpGSTs4). The fatty acid metabolism category had no representatives that showed differential changes in females related to the feeding treatment, and males lacked differentially expressed proteins in the transcription category.

## Discussion

### Overall male and female proteomes

Male and female protein functional category distributions were similar (Figure 1), despite only sharing ∼60% identity of specific proteins between them. The overall similar pattern of functional categories present in males and females may reflect the similar physiological challenges in both sexes during host colonization. An EST study of *Ips pini* midguts showed similar category distributions, with carbohydrate metabolism being highly represented, more so than in insects that do not feed on phloem [34]–[35]. Microarray data also found carbohydrate metabolism to be well represented in midguts [35].

Digestion of plant cell walls is of obvious importance to phytophagous animals. Plant cell wall degrading enzymes (PCWDEs) have been identified in the transcriptome and genome of MPB and other beetles [23] [36] [37]. Several PCWDEs were present in the MPB proteomes, but quantities did not change with host colonization. Dispersing beetles may maintain constitutive expression of PCWDEs. Both sexes maintained proteins with annotation matches to endopolygalacturonase, secreted cellulase, cellulose 1-4-β-cellobiosidase, and amylase A. Females had three pectinesterase representatives. The presence of PCWDEs affirms their importance during host colonization and indicates that colonizing beetles are already primed for wood digestion upon arriving at a suitable host tree.

### Distribution of ‘unique’ proteins

We identified differences between the functional category distributions of proteins uniquely presented in females or males. Because female MPB are the pioneering sex, an increase in carbohydrate metabolism and cell respiration may be necessary for producing the energy required to establish a maternal gallery, produce aggregation pheromone components, and increase provisioning to eggs while simultaneously overcoming host defences.

Males expressed proteins directly related to host colonization. One sub-category of constitutively expressed proteins unique to males were those involved in isoprenoid pheromone biosynthesis, including HMG-S. HMG-S is part of the mevalonate pathway, which synthesizes juvenile hormone (JH), a molecule that mediates numerous developmental changes related to differentiation and reproduction [38]. In *Ips pini*, HMG-S has been found to be inducible by JH, and the mevalonate pathway responds to feeding on host phloem [39]–[40]. Pheromone production is not exclusively dependent on phloem-derived precursors in some species; exposure to JH elicits pheromone production in *I. paraconfsus* and *I. pini* [41]–[43]. Several studies have demonstrated that JH induces the mevalonate pathway for synthesis of isoprenoid bark beetle pheromones such as frontalin, ipsenol and ipsdienol [44]–[46]. Male MPB produce the multifunctional isoprenoid-derived pheromone, frontalin, to signal that a host tree has succumbed to mass attack [47]. Frontalin is synthesized via the mevalonate pathway [44] [48]–[49], explaining the exclusive detection of HMG-S in host-colonizing males. Another isoprenoid pathway protein unique to the male proteome was similar to GGPPS, which functions in MPB frontalin synthesis [50]. Matching to the same full-length cDNA clone (DPO061_E16) as the protein described here, DponGGPPS transcripts were expressed primarily in the anterior midguts of feeding males with mates, with accumulation peaking after 24 hours. RNA interference of DponGGPPS reduced frontalin content to 9% of that found in controls [50].

### Proteomic shifts following feeding

In total, 23 and 29 proteins of the identified proteins significantly changed in quantity following feeding in females and males, respectively (FDR<0.2). The lowest FDR obtained was 0.06. A less stringent FDR cutoff was used in this experiment (as in reference [31]) compared to that of the recently published MPB larval proteome [25]. We were trying to capture a very small window –the expression changes occurring during the shift from dispersal to host colonization (represented by 24 hours), whereas the study of MPB larval proteomes had sample collection times separated by months and by different larval instars. When compared to the larvae, 54% and 60% of the female proteome was also present in the spring and fall libraries, respectively. Males had 54% and 58% similarity to spring and fall larvae. All categories were represented similarly in all four comparisons, with the highest percentages being for the unknown and carbohydrate metabolism proteins (15% and 14%, respectively), followed by those assigned to structure and translation (∼11%).

Generally, females had proteomic shifts of greater magnitude than the males. Seven proteins related to translation showed increases in females following feeding (fed males had one increase and one decrease), which could suggest a higher investment in translational machinery and explain the greater fold change values.

A transcriptome analysis of this sample set has also been conducted [51], and all but 3 proteins listed in Tables 1 and 2 matched transcripts detected in that study (identified by the Genome Accession numbers to which they were mapped). Those that were also found to significantly change with treatment in the transcriptome study have a fold change value marked with an asterisk. Gene expression analysis revealed many more significantly changing genes, which could be indicative of differences in isolation efficiency. For example, proteins range greatly in size and solubility and occupy different subcellular compartments. Both studies used the same sample pool and thus the same temporal ‘snapshot’; comparisons should bear in mind the time between transcription and translation and the influence of posttranscriptional and posttranslational modifications on the final protein product. That said, comparing these results where possible provides a more complete picture of what processes might be occurring.

### Chaperones and host colonization

Two proteins with BLAST matches to insect 70 kDa heat shock proteins (HSPs) increased significantly with host colonization in both females (3.4- and 2.8-fold change) and males (1.6- and 1.4-fold change). Both HSPs were also found to increase on the transcriptome level (YQE07729, [51]). Many stressors illicit a HSP response, including heat, desiccation, radiation, and chemical compounds [52]. HSPs are chaperones that facilitate the repair of misfolded or damaged enzymes. HSPs also assist in the proper folding of nascent proteins and are thought to control regulators of cellular signals for homeostasis, proliferation and apoptosis [53]. The significant increase in these proteins in both sexes highlights the role of stress physiology during host colonization. In fact, both proteins also exhibited a significant increase in MPB larvae during springtime [25], suggesting that these HSPs are very important to feeding MPB.

Further indication of stress physiology in host-attacking males is the presence of calreticulin – a chaperone – that exhibited one of the higher significant increases of proteins detected in the fed male proteome (1.5-fold change). Calreticulin increased by the same magnitude in MPB larvae as they developed in spring [25]. The transcript of this protein also increased in host-exposed males (YQE10054, [51]) and microarray analysis of *Ips pini* midgut tissue also indicated an induction with feeding [35]. Insect calreticulin has also been shown to be part of the response to microorganisms [54]. In some insects calreticulin may mediate phagocytosis of foreign bodies via an association with hemocytes as a part of the innate immune system. Exposure to the host, which will also be more susceptible to other pathogens as the beetles overwhelm its defensive systems, may require an enhanced immune response against an increasingly microorganism-laden environment. While calreticulin is present, it is unclear why females would not also increase production of this protein, but they do arrive at the host tree first. Males could be arriving at a time where secondary infection by microorganisms is advancing, making an enhanced immune system important for their initial contact with the host environment.

### Host colonization increases the abundance of detoxification enzymes

Several enzymes with potential functions in detoxification were differentially expressed in the female proteome. One upregulated protein was a cytochrome P450, a member of a ubiquitous group of enzymes known for their roles in detoxification, pheromone biosynthesis, and hormone metabolism [55]. First identified in the MPB EST library (AFI45031, [23]), CYP6DE1 increased by 1.8-fold with feeding. The corresponding transcript also showed an increase in the MPB transcriptome, although without statistical significance (YQE02814, [51]). CYP6DE1 was found to be decreasing in MPB larvae during the spring months; it was suggested that this was in response to host defenses waning as the tree succumbed to the attack [25].

The proteins with a relatively higher fold-change in fed males included one matching to the sigma GST (1.3-fold change), annotated DpGSTs4 [36]. GSTs are generally recognized as detoxification enzymes which conjugate toxins with glutathione, thereby facilitating their excretion [56]–[57]. DpGSTs4's expression pattern is consistent with a possible role in MPB coping with its toxic host environment.

### Host colonization and the initiation of reproduction

Proteins linked to reproductive physiology were shown to react quantitatively to feeding treatment in females. Two vitellogenin-matching proteins had significant and substantial fold change increases, specifically an 8.9-fold increase for vitellogenin-6 precursor and a 7.8-fold increase for vitellogenin. This pattern echoes the increases of vitellogenin transcripts in fed females (YQE09293, [51]). Vitellogenin, and its precursor, are phospholipoglycoprotein yolk precursors from the female's fat body that are taken up by maturing insect oocytes in a process controlled by JH [58]. Although it exceeded the 0.2 FDR cut-off, maternal exuperantia protein (Table S1 in File S1, h_cluster_01681-1+3) increased with feeding treatment (2.3-fold change, *p = 0.006*, FDR = 0.21). This protein – involved in promoting the transcription of reproductive genes – mediates the action of the bicoid gene product – a maternal effect gene vital for early larval development and the establishment of correct anatomical polarity [59]. A protein matching to hormone metabolism – a possible JH binding protein called soldier-specific protein 1 [60]– exhibited the largest fold-change decrease of the proteins detected in the female proteome (-2.5-fold change). The transcript for this protein was also found to decrease with feeding (YQE10157, [51]). JH is linked to pheromone production in some bark beetles and to reproduction and many other physiological processes in insects. That females wait until encountering the host tree and commencing with colonization activities to produce these reproductive proteins indicates a tradeoff between dispersal and reproduction.

This tradeoff and rapid upregulation of reproductive activity once dispersal is completed is also supported by indicators of females using host tissue as an energy source. Two proteins with BLASTx matches to components of a vacuolar H^+^-transporting ATPase – a complex involved in a variety of cellular processes related to active transport systems – increased in females with feeding (2.2- and 1.2-fold change). Fed *Manduca sexta* larvae have active transporter systems in the midgut, and the supply of K^+^ needed for digestion requires a gradient of H^+^ facilitated by V-type H^+^-transporting ATPases [61]. The increased production of these proteins in female MPB may similarly indicate the activation of gut tissue once feeding begins. Two carbohydrate metabolism proteins involved in glycolysis– transketolase and glucose-6-phosphate dehydrogenase – showed significant increases in females (2.4 and 1.3 fold-change) during early host colonization. This trend is corroborated, albeit not significantly, by the transcriptome data (YQE11922, YQE03281, [51]). Female MPB have been found to compensate for lower energetic stores after dispersal by obtaining energy at the breeding site (phloem) [16]. Here females are expressing proteins related to carbohydrate metabolism once they colonize a host, likely to process the phloem they consume during gallery excavation into energy. Combined with the production of reproductive proteins, this shift in energetics over just 24 hours demonstrates the speed and extent of the transition from flight dispersal to reproductive physiology in female MPB upon entering a host.

### Male muscle tissue as an energy source

Male MPB exhibited changes in proteins related to muscle and cellular structure. Females have higher relative lipid stores from which to draw energy [62], which might explain why we did not observe such changes in females. A protein matching to calponin increased significantly in colonizing males (1.4-fold change). Proteins in this family regulate muscle contractions, inhibiting the movement of actin filaments over myosin in vertebrate smooth muscle [63]. Insects exclusively have striated muscle, but calponin has been speculated to be integral to visceral muscle function [64]. Actinin exhibited a decrease with feeding treatment (-1.2-fold). Actinin cross-links actin filaments [65]–[66]. A protein that matched the light chain of kinesin, which is involved in cytoskeletal structuring, also decreased with feeding treatment (-1.3-fold change). This change in the way contractions are being regulated and the reduction of both muscle and cellular structure proteins may be related to males no longer needing their flight muscles, and it is likely that they begin to access muscle tissue for energy during early host colonization. As males do not construct the parental gallery, they may not be benefiting from energy contained in host phloem as females do [16].

## Conclusion

The proteomes of host colonizing males and females contained similar numbers of proteins, overall, some of which shifted significantly and rapidly following initial colonization of a pine host. In addition, the overall classification of proteins in both sexes resided in similar functional categories, but the proteins with quantities that significantly changed following feeding differed between the sexes.

Stress physiology played a role for both females and males during host colonization, as indicated by the significant accumulation of proteins such as various chaperones in both sexes as well as the CYP6DE1 in females and DpGSTs4 in males. Rapid accumulation of vitellogenin indicated that female reproductive physiology shifts dramatically during initial host colonization. Thus the mainly semelparous males and females – struggling in a highly variable and highly stressful environment during the initial hours of host colonization – rapidly shift towards and then prioritize reproductive physiology. MPB females conserve energy stores and then rapidly use those stores in their reproductive attempt, while males, conversely, consume much of their energy stores during the dispersal.

We detected indicators of different strategies between males and females during host colonization. Both sexes produced PCWDEs without feeding treatment, but had to encounter hosts before producing several chaperones and detoxification enzymes. However, males seemed to constitutively produce proteins related to pheromones, and hormones without host exposure. Females did not produce reproductive proteins until they colonized a host, at which point they also activated pathways relating to gut tissue transporters and carbohydrate metabolism. During colonization, males decreased proteins related to flight muscle, possibly to access energy. Females did not do this within the treatment time used here, suggesting that males and females employ different energetic strategies.

Not only were almost all of the differentially accumulating proteins discussed here detected in RNA-seq transcriptome analysis [51], but several transcripts with significant changes did so with the same patterns shown in our work. RNA-seq, microarray, and real-time PCR studies reveal the activity of genes through mRNA, but do not account for the effects of post-translational modification and regulation of the final protein products. This proteomics study provides another layer to the existing proteome, genome and transcriptome assets that have been developed for MPB [23] [25] [36] [51] [67] and provides new data and targets for ongoing exploration of the short, yet vital and perilous, host colonization phase of the MPB life cycle.

## Supporting Information

File S1 **Contains Tables S1, S2, and S3.** Table S1 - Female iTRAQ Run Data, Table S2 - Male iTRAQ Run Data, Table S3- Untranslated Transcriptome Sequences.
(XLSX)
